# Association of daytime napping in relation to risk of diabetes: evidence from a prospective study in Zhejiang, China

**DOI:** 10.1186/s12986-021-00545-4

**Published:** 2021-02-08

**Authors:** Hao Wang, Lingli Chen, Dun Shen, Yuan Cao, Xiaoyi Zhang, Kaixu Xie, Chunmei Wang, Shuiqing Zhu, Yu Guo, Bragg Fiona, Min Yu, Zhengming Chen, Liming Li

**Affiliations:** 1 Department of NCDs Control and Prevention, Zhejiang Provincial Center for Diseases Control and Prevention, #3399 Binsheng Road, Binjiang District, Hangzhou, Zhejiang Province China; 2 Department of NCDS Control and Prevention, Tongxiang City Center for Disease Control and Prevention, Tongxiang, China; 3 Chinese Academy of Medical Sciences, Beijing, China; 4 Medical Research Council Population Health Research Unit, Nuffield Department of Population Health, University of Oxford, Oxford, UK; 5 Department of Epidemiology and Biostatistics, School of Public Health, Peking University, Beijing, China

**Keywords:** Daytime napping, Diabetes, Prospective study, China

## Abstract

**Background:**

Diabetes was a major risk factor for numerous chronic diseases. However, the associations between daytime napping and diabetes in the existing literature is still inconsistent.

**Methods:**

The analysis included 53,916 participants aged 30–79 years of the China Kadoorie Biobank prospective study from Tongxiang. Incident diabetes were identified through linkage with incident diabetes surveillance systems, health insurance system, and death registries. Cox regressions were used to estimate the associations of daytime napping with diabetes.

**Results:**

5.11% of participants reported habitual daytime napping. During 488,233 person-years (median 9.4 years) of follow-up, 3333 incident diabetes, including 1249 males and 2084 females, were documented. After adjusting for socio-demographic status, behavioral lifestyle, BMI, waist circumference and snoring, as comparison with those without daytime napping, the hazard ratios for risk of diabetes were 1.39 (95% CI 1.21–1.59). The corresponding figures for males and females were 1.45 (95% CI 1.20–1.74) and 1.30 (95% CI 1.05–1.59), respectively. The corresponding figures for postmenopausal and premenopausal females were 1.41 (95% CI 1.08–1.80) and 1.13 (95% CI 0.78–1.59), respectively.

**Conclusions:**

Habitual daytime napping is positively associated with risk of diabetes in adults, except premenopausal females.

## Background

Diabetes was a major risk factor for numerous chronic diseases, including heart disease, ischemic stroke and renal disease [1, 2]. Nearly 463 million adults aged 20–79 years had diabetes worldwide, of whom three quarters lived in low and middle income countries [3]. China had the highest number of diabetics, which will continue to increase [3, 4]. A nationally representative survey indicated that the prevalence of diabetes was 10.9% among adults over 18 years in China [5]. The estimated cost of diabetes in 2015 was 1.3 trillion dollars, accounting for approximately 1.8% of global gross domestic product [6].

Daytime napping was defined as a short sleep typically taken in the early afternoon [7]. It was one of the common behavioral habits worldwide, especially among Chinese adults. In China, napping is thought as part of healthy lifestyle and has been deeply embedded into the culture [8]. The benefit of napping included sleepiness reduction, memory consolidation, cognitive performance enhancement, boost in emotional stability, and endurance performance improvement [9]. 20.3% of 30–79 years old adults are reported to be habitual napper in China [10]. However, the association of daytime napping with diabetes from published studies remain inconsistent. While one prospective cohort study suggested that daytime napping was associated with an increased diabetes risk in British population [11], another prospective study indicated that null significant association was found in Finnish population [12]. Hence, the purpose of this study was to examine the association of daytime napping with risk of incident diabetes using data from Tongxiang Zhejiang in the China Kadoorie Biobank.

## Methods

### Study population and design

CKB study is a nationwide, prospective cohort study. Detailed information on the CKB study design, method, and participants have been reported previously [13–15]. The data utilized in the present study came from Tongxiang, one of the 10 regions in China. Briefly, 57704 participants aged 30–79 years old were recruited and participated in the baseline survey between August 2004 and January 2008.

### Assessment of daytime napping

Daytime napping was assessed through the question “Do you usually take a daytime nap?” Answer options included “Yes usually”, “Yes, but only in summer”, and “None”. Participants who chose the first option were defined as habitual daytime nappers. Participants who chose the third option were defined as non-nappers.

### Assessment of covariates

Detailed covariates information was collected at baseline by means of a standardized questionnaire, including sociodemographic characteristics (age, sex, education level, marital status, and household income), lifestyle behaviors (cigarette consumption, alcohol consumption, physical activity, intakes of fresh fruit, vegetable and meat, sleep duration and snoring), personal medical history (cancer, stroke, diabetes, and heart attack), family history of diabetes, and women menopause status. Height, weight, and waist circumference (WC) were measured using calibrated instruments by trained staff.

Standing height was measured to the nearest 0.1 cm with a stadiometer. Weight was measured to the nearest 0.1 kg with a body composition analyzer (TANITA-TBF-300GS), subtracting the weight of clothing according to season (0.5 kg in summer and 2.0–2.5 kg in winter). Body mass index (BMI) was calculated as weight in kilograms divided by the square of height in meters, and obesity was defined as BMI ≥ 25.0 kg/m^2^ [16]. WC was measured to the nearest 0.1 cm with a non-stretchable tape measure. WC was measured at the midpoint between the lowest rib margin and the iliac crest. A non-fasting venous blood sample was collected. Immediate on-site testing of plasma glucose level was undertaken. Random blood glucose levels were measured using the SureStep Plus System (Johnson & Johnson, New Brunswick, NJ, USA) Participants who did not report a history of diabetes but who had random blood glucose ≥ 11.1 mmol/l or fasting blood glucose ≥ 7.0 mmol/l were defined as having screen-detected diabetes.

In the present study, participants with self-reported history of physician-diagnosed chronic diseases (163 with cancers, 349 with strokes, 464 with heart diseases and 1380 with diabetes) and 1432 participants with screen-detected diabetes at baseline, were excluded, yielding a total of 53 916 (22,573 men, 31,343 women) participants in the final analysis.

### Follow-up for incident diabetes

All the participants were followed up from the date of baseline survey until the date of diabetes events, death, or 31 December 2015. Incident diabetes were obtained periodically through linkage with local diabetes surveillance system, death registries, and with national insurance electronic system, covering more than 98% study participants in Tongxiang. Trained staff, blinded to the baseline information, coded all cases with the 10th revision of International Classification of Diseases (ICD-10). For the present analysis, we included diabetes cases as E10–E14.

### Statistical analysis

Percentage and mean values of characteristics at baseline were calculated according to answer of daytime napping. Cox proportional hazards regression was used to estimate adjusted hazard ratios (HRs) of diabetes in relation to daytime napping and 95% confidence intervals. Four different models were performed to calculate HRs of diabetes risk. Models were stratified by age at baseline (in 5-year intervals), and sex. In model 1, HRs were adjusted for age and sex. In model 2, HRs was further adjusted for education level (no formal education, primary school, middle school, and high school or above), household income (< 19,999 yuan, 20,000–34,999 yuan, ≥ 35,000 yuan), marital status, cigarettes consumption (never, occasional, former, and current regular), alcohol consumption (never, occasional, former, and current regular), meat consumption (daily and non-daily), fresh fruits consumption (daily and non-daily), fresh vegetables consumption(daily and non-daily), physical activity (metabolic equivalent of tasks hours/day; continuous), and sleep duration (continuous). In model 3, HRs were further adjusted for BMI (continuous) and WC (continuous). In model 4, HRs were further adjusted for self-reported snoring (none, occasional, and habitual). In subgroup analyses, participants were divided into non-obese group and obese group according to their BMI, and females were divided into premenopausal group and post-menopausal group according to their menopause status. In sensitivity analyses, the first 2 years of follow-up was excluded to reduce the effect of reverse causality, and those with diabetes family history was excluded to reduce the effect of family history. All statistical analysis was calculated by using SAS version 9.3, and a two-sided *P* < 0.05 was defined as statistically significant.

## Results

### Characteristics of participants

Of the 53 916 participants included, the mean (SD) baseline age was 52.0 (9.9) years and 58.1% was female. 37.8% of participants' household income were more than 35,000 (yuan). 28.0% of participants were current regular smokers. 17.1% of participants were current regular drinkers. The average of total physical activity of participants was 30.6 MET-h/d. 15.2% of participants consumed meat daily. 6.7% of participants consumed fresh fruits daily. 93.8% of participants consumed vegetables daily. The average of BMI was 22.9 kg/m^2^ and the average of WC was 76.5 cm. The average of sleep duration of participants was 7.6 h daily. 53.7% of women was postmenopausal. As compared with non-nappers, habitual nappers were more likely to be older, to be males, to be well-educated, to have more household income, to be current regular drinker, to be physically inactive, to consume meat and fruit frequently, to have a higher BMI and WC, and to have more sleep duration, and less likely to be current regular smokers. No significant difference of the proportion of postmenopausal females between three groups. (*P* = 0.86). (Table 1).

**Table 1:** Baseline characteristics of the participants by frequency of daytime napping

Characteristics	Overall (n = 53 916)	Daytime napping			*P*
		Habitual (n = 2757)	Only in summer (n = 17,648)	Non-napping (n = 33,511)	
Mean age (years)	52.0 ± 9.9	53.1 ± 10.8	52.9 ± 9.8	51.3 ± 9.7	< 0.0001
Females (%)	58.1	43.1	51.0	63.2	< 0.0001
High school or above (%)	3.9	6.7	4.5	3.4	< 0.0001
Married (%)	93.1	93.1	93.5	92.8	0.0008
Household income ≥ 35,000 (yuan) (%)	37.8	40.9	38.7	37.0	< 0.0001
Current smokers (%)	28.0	26.7	27.6	28.5	< 0.0001
Current drinkers (%)	17.1	17.9	17.7	16.7	0.0011
Physical activities (MET-h/d)	30.6 ± 15.3	26.4 ± 16.3	29.3 ± 14.9	31.6 ± 15.1	< 0.0001
Consuming meat daily (%)	15.2	26.9	16.1	13.8	< 0.0001
Consuming fruit daily (%)	6.7	16.7	7.8	5.5	< 0.0001
Consuming vegetable daily (%)	93.8	93.7	92.9	94.3	< 0.0001
BMI (kg/m^2^)	22.9 ± 3.1	23.2 ± 3.3	23.0 ± 3.1	22.8 ± 3.1	< 0.0001
WC (cm)	76.5 ± 9.1	77.7 ± 9.6	76.7 ± 9.1	76.3 ± 8.9	< 0.0001
Sleep duration (hours)	7.6 ± 1.2	8.0 ± 1.3	7.8 ± 1.2	7.5 ± 1.1	< 0.0001
Postmenopausal (%)^a^	53.7	53.8	53.7	53.7	0.86

Data were adjusted for age and sex, as appropriate

*MET* metabolic equivalent tasks, *BMI* Body Mass Index, *WC* waist circumference

^a^Only among the females

### Percentage of daytime napping

Of all participants, the proportion of habitual daytime napping, napping only in summer, and non-napping were 5.11%, 32.72% and 62.15%, respectively. The percentage of habitual daytime napping among 30–49 years, 50–59 years, and 60–79 years group were 4.49 (95% CI 4.22–4.75), 4.63 (95% CI 4.33–4.93), and 7.02 (95% CI 6.57–7.48), respectively. The percentage of habitual daytime napping increased with an increase of age (*P* < 0.0001). The percentage of habitual daytime napping was higher among males than females (7.17% vs. 3.63%) (*P* < 0.0001). (Table 2).

**Table 2:** Percentage of habitual daytime napping by different characteristics

Characteristics	Participants	Habitual daytime napping		x^2^	*P*
		n	Percentage (95% CI)		
Age range (years)				89.61	< 0.0001
30–49	23,099	1036	4.49 (4.22–4.75)		
50–59	18,516	857	4.63 (4.33–4.93)		
60–79	12,301	864	7.02 (6.57–7.48)		
Sex				339.20	< 0.0001
Males	22,573	1619	7.17 (6.89–7.45)		
Females	31,343	1138	3.63 (3.46–3.80)		

### Association of daytime napping with diabetes

During 488,233 person-years (median 9.4 years) of follow-up, 3333 incident diabetes, 1249 were males, and 2084 were females were documented. For habitual daytime napping, after adjusting for socio-demographic status, cigarettes, alcohol, physical activity, dietary consumption and sleep duration, in comparison with non-nappers, the adjusted HRs for risk of diabetes were 1.55 (95% CI 1.35–1.77). The corresponding figures for males and females were 1.67 (95% CI 1.38–2.01) and 1.43 (95% CI 1.16–1.75), respectively. After additional adjustment for BMI and WC, the corresponding HRs for risk of incident diabetes among all of the participants were attenuated to be 1.41 (95% CI 1.23–1.62). The corresponding figures for males and females were 1.47 (95% CI 1.22–1.76) and 1.33 (95% CI 1.08–1.63), respectively. After additional adjustment for self-reported snoring, habitual nappers had a 39% higher risk of diabetes than non-nappers. Similar positive associations were found among males (HR = 1.45, 95% CI 1.20–1.74) and females (HR = 1.30, 95% CI 1.05–1.59). (Table 3).

**Table 3:** Adjusted hazard ratios for diabetes in relation to daytime napping among participants of Zhejiang

	N. of participants	N. of incident diabetes	Model 1	Model 2	Model 3	Model 4
			HR (95% CI)	HR (95% CI)	HR (95% CI)	HR (95% CI)
Total						
Non-napping	33,511	1942	1	1	1	1
Napping only in summer	17,648	1145	1.11 (1.04–1.20)	1.09 (1.01–1.18)	1.06 (0.99–1.14)	1.05 (0.97–1.13)
Habitual daytime napping	2757	246	1.62 (1.42–1.85)	1.55 (1.35–1.77)	1.41 (1.23–1.62)	1.39 (1.21–1.59)
Males						
Non-napping	12,265	599	1	1	1	1
Napping only in summer	8689	505	1.16 (1.03–1.31)	1.13 (1.01–1.28)	1.09 (0.96–1.23)	1.07 (0.95–1.21)
Habitual daytime napping	1619	145	1.81 (1.50–2.16)	1.67 (1.38–2.01)	1.47 (1.22–1.76)	1.45 (1.20–1.74)
Females						
Non-napping	21,246	1343	1	1	1	1
Napping only in summer	8959	640	1.09 (0.99–1.20)	1.08 (0.98–1.18)	1.05 (0.95–1.15)	1.03 (0.93–1.13)
Habitual daytime napping	1138	101	1.45 (1.17–1.76)	1.43 (1.16–1.75)	1.33 (1.08–1.63)	1.30 (1.05–1.59)

Model 1, adjusted for age and sex. Model 2, further adjusted for education level (no formal education, primary school, middle school, and high school or above), household income (< 19,999 yuan, 20,000–34,999 yuan, and ≥ 35,000 yuan), marital status, cigarettes consumption (never, occasional, former, and current regular), alcohol consumption (never, occasional, former, and current regular), meat, fresh fruits, and fresh vegetables consumption (daily and non-daily), physical activity (continuous) and sleep duration (continuous). Model 3, further adjusted for and BMI (continuous) and WC (continuous). Model 4, further adjusted for snoring (none, occasional, and habitual)

For participants who nap only in summer, after adjusting for socio-demographic status, cigarettes, alcohol, physical activity, dietary consumption, sleep duration, BMI and WC, in comparison with non-nappers, the adjusted HRs for risk of incident diabetes were 1.06 (95% CI 0.99–1.14). The corresponding figures for males and females were 1.09 (95% CI 0.96–1.23) and 1.05 (95% CI 0.95–1.15), respectively. After additional adjustment for self-reported snoring, similar non-significant associations were found among all participants (HR = 1.05, 95% CI 0.97–1.13), males (HR = 1.07, 95% CI 0.95–1.21), and females (HR = 1.03, 95% CI 0.93–1.13) (Table 3).

### Subgroup analyses

Among non-obese participants, after adjusting for socio-demographic status, behavior lifestyle, BMI, WC, and self-reported snoring, habitual nappers had a 44% higher risk of incident diabetes than non-nappers (95% CI 1.18–1.75). The corresponding figures for obese participants was 1.35 (95% CI 1.11–1.63). No multiplicative interaction was found between habitual napping and obesity measures (P > 0.05) (Table 4**).**

**Table 4:** Adjusted hazard ratios for diabetes in relation to daytime napping among non-obese and obese participants

	N. of participants	N. of incident diabetes	Model 1	Model 2	Model 3	Model 4
			HR (95% CI)	HR (95% CI)	HR (95% CI)	HR (95% CI)
Non-obese (BMI < 25 kg/m^2^)						
Non-napping	25,470	1027	1	1	1	1
Napping only in summer	13,217	582	1.09 (0.98–1.20)	1.08 (0.97–1.20)	1.07 (0.97–1.19)	1.06 (0.95–1.17)
Habitual daytime napping	2032	121	1.56 (1.28–1.87)	1.54 (1.26–1.86)	1.47 (1.20–1.78)	1.44 (1.18–1.75)
Obese (BMI ≥ 25 kg/m^2^)						
Non-napping	8041	915	1	1	1	1
Napping only in summer	4431	563	1.08 (0.97–1.20)	1.06 (0.95–1.18)	1.06 (0.95–1.18)	1.04 (0.93–1.16)
Habitual daytime napping	725	125	1.47 (1.21–1.77)	1.43 (1.18–1.73)	1.38 (1.13–1.66)	1.35 (1.11–1.63)

Model 1, adjusted for age and sex. Model 2, further adjusted for education level (no formal education, primary school, middle school, and high school or above), household income (< 19,999 yuan, 20,000–34,999 yuan, and ≥ 35,000 yuan), marital status, cigarettes consumption (never, occasional, former, and current regular), alcohol consumption (never, occasional, former, and current regular), meat, fresh fruits, and fresh vegetables consumption (daily and non-daily), physical activity (continuous) and sleep duration (continuous). Model 3, further adjusted for and BMI (continuous) and WC (continuous). Model 4, further adjusted for snoring (none, occasional, and habitual)

Among postmenopausal females, after adjusting for socio-demographic status, behavior lifestyle, BMI, WC, and self-reported snoring, habitual daytime nappers had a 41% higher risk of diabetes than non-nappers (95% CI 1.08–1.80). However, null statistically significant association was found among premenopausal females (HR = 1.13, 95% CI 0.78–1.59). (Table 5).

**Table 5:** Adjusted hazard ratios for diabetes in relation to daytime napping among postmenopausal and premenopausal females

	N. of participants	N. of incident diabetes	Model1	Model2	Model3	Model4
			HR (95% CI)	HR (95% CI)	HR (95% CI)	HR (95% CI)
Postmenopausal females						
Non-napping	11,047	849	1	1	1	1
Napping only in summer	5192	443	1.12 (0.99–1.25)	1.10 (0.98–1.24)	1.07 (0.95–1.20)	1.04 (0.93–1.17)
Habitual daytime napping	581	67	1.55 (1.20–1.97)	1.50 (1.15–1.92)	1.44 (1.11–1.85)	1.41 (1.08–1.80)
Premenopausal females						
Non-napping	10,199	494	1	1	1	1
Napping only in summer	3767	197	1.03 (0.87–1.21)	1.03 (0.87–1.21)	1.02 (0.86–1.21)	1.01 (0.85–1.20)
Habitual daytime napping	557	34	1.27 (0.88–1.77)	1.28 (0.88–1.79)	1.16 (0.80–1.62)	1.13 (0.78–1.59)

Model 1, adjusted for age. Model 2, further adjusted for education level (no formal education, primary school, middle school, and high school or above), household income (< 19,999 yuan, 20,000–34,999 yuan, and ≥ 35,000 yuan), marital status, cigarettes consumption (never, occasional, former, and current regular), alcohol consumption (never, occasional, former, and current regular), meat, fresh fruits, and fresh vegetables consumption (daily and non-daily), physical activity (continuous) and sleep duration (continuous). Model 3, further adjusted for and BMI (continuous) and WC (continuous). Model 4, further adjusted for snoring (none, occasional, and habitual)

### Sensitivity analyses

The associations of habitual daytime napping with diabetes did not changed appreciably after exclusion of the first 2 years of follow-up to reduce the effect of reverse causality, exclusion of those with diabetes family history to reduce the effect of family history. (Additional file 1: Table S1).

## Discussion

In this large prospective study, the association of daytime napping with incident diabetes was explored. Habitual daytime napping was observed to be positively associated with risk of incident diabetes. However, such positive association was not found among premenopausal females.

### Percentage of habitual daytime napping

The percentage of habitual daytime napping in our study was 5.11%, much lower than the average level of CKB in ten study regions (20.3%). This difference reflected geographic diversity on distribution of daytime napping in China [10]. Consistent with the previous studies [10, 17], the percentage of habitual daytime napping increased with increasing age. Similar with the previous studies [7, 10, 17], the percentage of habitual daytime napping was higher in males than in females in the present study.

### Association of daytime napping with diabetes

One meta-analysis, including 304,885 individuals from four cross-sectional and six longitudinal studies, indicated that person with daytime napping had higher risk of diabetes in both cross-sectional and cohort studies than those without daytime napping [18], which was compatible with the present study. Another meta-analysis of 249,077 participants and 13,237 incident diabetes, comprising 7 prospective studies (one from the USA, two from China, and four from Europe), found that person with habitual daytime napping were 17% more likely to develop diabetes in comparison with those without daytime napping (RR = 1.17, 95% CI 1.08–1.27). By region, while relative risks (95% CI) of diabetes for the USA, and Europe were 1.21 (1.17–1.26) and 1.15 (1.03–1.30), respectively. No significant association of daytime napping with diabetes was found among Chinese adults (RR = 1.23, 95% CI 0.87–1.73) [19].

Contrary to one previous study performed in Guangzhou indicating that no significant association of daytime napping with prevalent diabetes was identified among Chinese males [17], the present study demonstrated that males with habitual daytime napping had a 45% higher risk for incident diabetes compared with the counterparts without napping after adjustment for socio-demographic status, behavioral lifestyle, BMI, WC, and self-reported snoring.

Previous studies reported that the effect of obesity on association of daytime napping with risk of diabetes was controversial. Hublin et al. found that the association of habitual nappers was associated with an increased risk for diabetes [12], but this association become non-significant after further adjustment for BMI, and concluded that napping is not an independent risk for diabetes. However, other studies, conducted in UK [11], USA [20], and China [21] showed that associations of habitual daytime napping with diabetes after adjustment for BMI were somewhat attenuated, but still statistically significant, consistent with our results. Health ABC study showed that diabetic patients had higher odds of daytime napping compared to those without diabetes [22], and concluded that daytime napping might be a consequence of diabetes. In the current study, both self-reported physician-diagnosed diabetes and screen-detected diabetes at baseline were excluded from the final analysis, which ensured a temporal sequence between daytime napping and diabetes.

### Subgroup analysis

To better observe the effect of obesity on the association of daytime napping with diabetes, analysis were conducted in both non-obese and obese population group separately. Notably, association between habitual daytime napping and risk of incident diabetes was significant not only in obese population group, but also in non-obese population group. This findings address the importance of reducing daytime napping among population with normal weight. Furthermore, because obesity has been widely accepted as an important risk for diabetes, our findings of significant association in non-obese population imply that besides obesity, there are other possible mechanisms explaining association of daytime napping with incident diabetes.

Menopause was associated with an adverse metabolic profile that increase the risk of diabetes [23]. However, few studies have observed the association of daytime napping with diabetes among premenopausal and postmenopausal women. One cross-sectional study including 6940 females aged 45 years or older from China indicated middle-aged postmenopausal women with more than 60 daytime napping per day had an 81% higher risk of diabetes in comparison with those without daytime napping. No significant differences were found among middle-aged premenopausal women [24], which was in line with our findings.Future prospective studies are needed to explore the association of habitual daytime napping with diabetes in premenopausal women.

### Possible mechanisms

There are several possible mechanisms explaining causal association of daytime napping with increased risk of diabetes. First, long daytime napping might disturb circadian rhythms [25], with increased night awakening and reduced nocturnal sleep duration [26], and subsequently, caused insulin resistance and diabetes [27, 28]. Additionally, daytime napping was recognized as a compensation for sleep loss or poor sleep quality due to increasing sleep fragmentation resulting from sleep disorders [29]. Sleep loss and poor sleep quality might contribute to the development of diabetes [30, 31]. Nevertheless, the association between daytime napping and nocturnal sleep was not completely identical. Previous studies indicated that duration of daytime napping appeared to be irrelevant to neither duration of nocturnal sleep nor its quality [20, 32]. Hence, association of daytime sleep with nocturnal sleep are needed to be explored in the future. Second, daytime napping was positively associated with inflammatory markers, including serum C reactive protein [33], which might enhance the development of diabetes [34, 35]. Third, daytime napping was an indicator of obstructive sleep apnea (OSA) [36]. OSA is characterized by the recurrent collapse of respiratory structures [37], which could increase sympathetic nervous activity and oxidative stress, and eventually cause insulin resistance [38]. Lastly, long daytime napping might decrease physical activity, resulting obesity and diabetes [39]. This assumption was also proven in our study that participants with habitual daytime napping had much less physical activity than non-nappers.

### Strengths and limitations

The strength of this study include a large sample size, a prospective study design, and a relatively long follow-up duration. The exclusion of diagnosed and undiagnosed diabetes at baseline reduced the potential of reverse causality. The study also has several limitations. First, assessment of daytime napping was qualitative, lacking information on napping duration in questionnaire, restricts the ability to further explore association of short and long daytime napping duration with risk of incident diabetes. One previous study indicated that short daytime napping (< 60 min/day) was not related to diabetes, and long napping was significantly associated with an increased risk of diabetes [40]. Second, although several established and potential risk factors for incident diabetes were adjusted for in different models, residual confounding by other unmeasured or unknown biological and social factors was still possible.

## Conclusions

In conclusion, the current study presents that habitual daytime napping is positively related to diabetes in Chinese adults, except premenopausal females.

## Supplementary information

**Additional file 1: Table S1.** Adjusted hazard ratios for diabetes in relation to daytime napping among specific participants.

## Data Availability

Details of how to access China Kadoorie Biobank data and details of the data release schedule are available from www.ckbiobank.org/site/ Data + Access.

## Abbreviations

CKB: China Kadoorie Biobank
BMI: Body mass index
WC: Waist circumference
ICD: International classification of diseases
HRs: Hazard ratios
