# Supplementary material for: Association of daytime napping in relation to risk of diabetes: evidence from a prospective study in Zhejiang, China
Source: Nutr Metab (Lond). 2021 Feb 8;18:18. doi: 10.1186/s12986-021-00545-4 (PMC7869458; doi:10.1186/s12986-021-00545-4)
Supplement: Supplementary file 1 — Additional file 1: Table S1. Adjusted hazard ratios for diabetes in relation to daytime napping among specific participants. [file 12986_2021_545_MOESM1_ESM.docx]

**Table S1 Adjusted hazard ratios for diabetes in relation to daytime napping among specific participants**

|  | N. of participants | N. of incident diabetes | Model1 | Model2 | | Model3 | Model4 |
| --- | --- | --- | --- | --- | --- | --- | --- |
|  |  |  | HR (95%CI) | HR (95%CI) | | HR (95%CI) | HR (95%CI) |
|  |  |  | **Exclude first 2 years follow-up** | | | | |
| Non-napping | 32 867 | 1 532 | 1 | 1 | | 1 | 1 |
| Napping only in summer | 17 291 | 944 | 1.17 (1.08-1.27) | 1.13 (1.04-1.22) | | 1.09 (1.01-1.19) | 1.08 (0.99-1.17) |
| Habitual daytime napping | 2 670 | 199 | 1.69 (1.45-1.95) | 1.54 (1.32-1.78) | | 1.40 (1.20-1.63) | 1.37 (1.18-1.59) |
|  |  |  | **Exclude those with diabetes family history** | | | | |
| Non-napping | 32 400 | 1 804 | 1 | | 1 | 1 | 1 |
| Napping only in summer | 17 019 | 1 065 | 1.12 (1.04-1.21) | | 1.10 (1.02-1.19) | 1.07 (0.99-1.16) | 1.06 (0.98-1.14) |
| Habitual daytime napping | 2 645 | 233 | 1.66 (1.45-1.91) | | 1.59 (1.38-1.83) | 1.45 (1.26-1.67) | 1.43 (1.24-1.64) |

Model 1, adjusted for age and sex. Model 2, further adjusted for education level (no formal education, primary school, middle school, and high school or above), household income (<19 999 yuan, 20 000-34 999 yuan, and ≥35 000 yuan), marital status, cigarettes consumption (never, occasional, former, and current regular), alcohol consumption (never, occasional, former, and current regular), meat, fresh fruits, and fresh vegetables consumption (daily and non-daily), physical activity (continuous) and sleep duration (continuous). Model 3, further adjusted for and BMI (continuous) and WC (continuous). Model 4, further adjusted for snoring (none, occasional, and habitual).
